# IRS-2 deubiquitination by USP9X maintains anchorage-independent cell growth via Erk1/2 activation in prostate carcinoma cell line

**DOI:** 10.18632/oncotarget.26049

**Published:** 2018-09-21

**Authors:** Haruka Furuta, Hidehito Yoshihara, Toshiaki Fukushima, Yosuke Yoneyama, Akihiro Ito, Claire Worrall, Ada Girnita, Leonard Girnita, Minoru Yoshida, Tomoichiro Asano, Masayuki Komada, Naoyuki Kataoka, Kazuhiro Chida, Fumihiko Hakuno, Shin-Ichiro Takahashi

**Affiliations:** ^1^ Departments of Animal Sciences and Applied Biological Chemistry, Graduate School of Agriculture and Life Sciences, The University of Tokyo, Tokyo, Japan; ^2^ Department of Medical Science, Graduate School of Medicine, Hiroshima University, Hiroshima, Japan; ^3^ Cell Biology Center, Institute of Innovative Research, Tokyo Institute of Technology, Yokohama, Japan; ^4^ Chemical Genomics Research Group, RIKEN Center for Sustainable Resource Science, Saitama, Japan; ^5^ Department of Oncology and Pathology, Cancer Center Karolinska, Karolinska Institute and Karolinska University Hospital, Stockholm, Sweden; ^6^ Present address: Institute of Research, Tokyo Medical and Dental University, Tokyo, Japan

**Keywords:** ubiquitin, USP9X, IGF-I, IRS-2, prostate cancer

## Abstract

Insulin-like growth factors (IGFs) have been shown to induce proliferation of many types of cells. Insulin receptor substrates (IRSs) are major targets of IGF-I receptor (IGF-IR) tyrosine kinase activated by IGFs, and are known to play important roles in the activation of downstream signaling pathways, such as the Erk1/2 pathway. Dysregulation of IGF signaling represents a central tumor promoting principle in human carcinogenesis. Prostate carcinoma is highly dependent on the IGF/IGF-IR/IRS axis. Here we identified the deubiquitinase, ubiquitin specific peptidase 9X (USP9X) as a novel binding partner of IRS-2. In a human prostate carcinoma cell line, small interfering RNA (siRNA)-mediated knockdown of USP9X reduced IGF-IR as well as IRS-2 protein levels and increased their ubiquitination. Knockdown of USP9X suppressed basal activation of the Erk1/2 pathway, which was significantly restored by exogenous expression of IRS-2 but not by IGF-IR, suggesting that the stabilization of IRS-2 by USP9X is critical for basal Erk1/2 activation. Finally, we measured anchorage-independent cell growth, a characteristic cancer feature, by soft-agar colony formation assay. Knockdown of USP9X significantly reduced anchorage-independent cell growth of prostate carcinoma cell line. Taken all together, our findings indicate that USP9X is required for the promotion of prostate cancer growth by maintaining the activation of the Erk1/2 pathway through IRS-2 stabilization.

## INTRODUCTION

Insulin-like growth factors (IGFs) induce a variety of bioactivities, such as growth, anti-apoptosis and differentiation in many types of cells [1]. IGF bioactivities are mediated mainly by insulin receptor substrate (IRS)-1 and IRS-2. In response to IGFs, IRS-1/2 are rapidly phosphorylated on multiple tyrosine residues by the activated IGF-IR kinase. The tyrosine-phosphorylated IRS-1/2 is recognized by the Src homology 2 (SH2) domain-containing molecules, including phosphatidylinositol 3-kinase (PI3K) and Grb2. By binding to IRS-1/2, PI3K is activated and generates phosphatidylinositol (3,4,5)-triphosphate (PIP3), resulting in Akt kinase activation. The recruitment of Grb2 and Sos to IRS proteins leads to the activation of guanine nucleotide exchange factor Sos, which in turn activates RAS and triggers the RAS/Raf/mitogen-activated-protein kinase/ERK kinase (MEK)/mitogen-activated protein kinase (MAPK) cascade, resulting in the extracellular-signal-regulated kinase (Erk1/2) activation. The activation of the Akt and Erk pathways, is crucial to the promotion of a variety of IGF-I bioactivities.

Earlier publications have shown that IGF-I signaling mediates carcinogenesis, malignant cell proliferation and metastatic growth of a variety of cancers [2–4]. IGF-IR is commonly expressed in human cancers, and it is well established that its expression level is highly associated with poor prognosis [2]. IRS-1/2 are also ubiquitously expressed in many types of cancer, and suggested to have roles in cancer initiation and progression. For example, overexpression of either IRS-1 or IRS-2 results in transformation in human MCF10A cells [5]. Similarly, transgenic expression of IRS-1 or IRS-2 in mouse mammary gland causes mammary hyperplasia and tumorigenesis, accompanied by the upregulation of Akt and Erk1/2 pathways [5, 6]. In addition, depletion of IRS-2 significantly diminished tumor growth and metastasis in a variety of mouse organs [7, 8]. These evidences clearly suggest IRS-1/2 as positive regulators of cancer features.

The growth of prostate cancer is highly dependent on IGF signaling [9–11], and over-activation of IGF signaling results in transformation in prostate epithelial cells [12]. It has been also reported that in human prostate tumor and several prostate cancer cell lines including PC3, the expression of IRS-2 is elevated [8]. These data support the idea that the highly expressing IRS-2 increases the activity of IGF signaling, and thereby has a role in the establishment and the maintenance of prostate cancer.

It is well known that ubiquitination plays an important role in the regulation of quantities of IRS-1/2. Several E3 ubiquitin ligases have been shown to ubiquitinate IRS-1/2 leading to their proteasomal degradation [13–17]. Furthermore, we have recently reported that a deubiquitinase, USP7 deubiquitinates IRS-1/2, resulting in their stabilization [18]. These findings indicate that deubiquitination as well as ubiquitination plays an important role in regulation of IRS-1/2 quantity. In the present study, we identified another deubiquitinase, USP9X as a novel IRS-associated protein and demonstrated that USP9X prevents ubiquitination and degradation of IRS-2. In addition, by using PC3 human prostate cancer cells, we revealed that IRS-2 stabilization by USP9X is required for the constitutive activation of Erk1/2. Furthermore, knockdown of either USP9X or IRS-2 impaired cell proliferation and anchorage-independent growth of PC3 cells.

## RESULTS

### USP9X interacts with IRS-1/2

**Figure 1:**
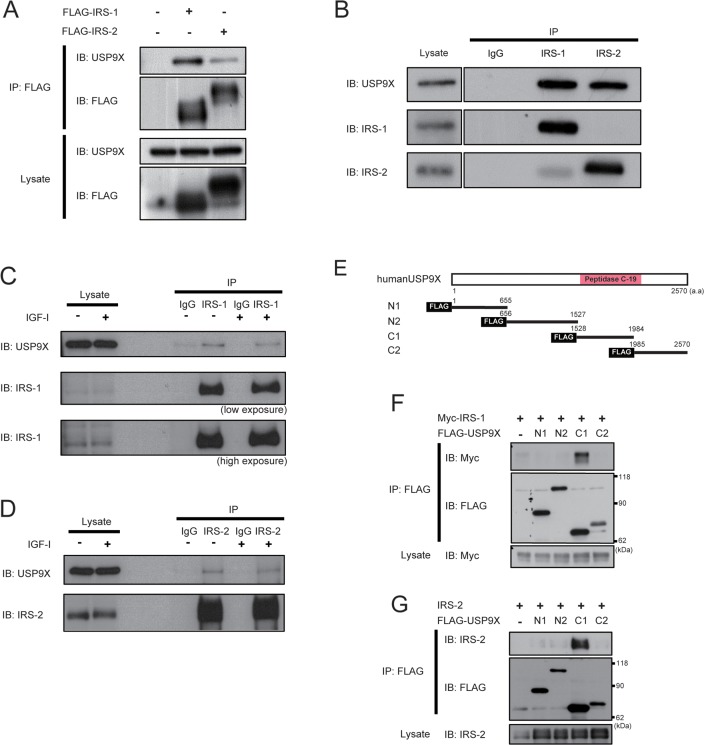
USP9X interacts with IRS-1/2 **(A)** HEK293 cells expressing FLAG-IRS-1, IRS-2 or FLAG alone were cultured under serum-free conditions for 24 hours. The cell lysates were immunoprecipitated with anti-FLAG antibody, followed by western blotting using indicated antibodies. **(B)** PC3 cells were serum-starved for 24 hours, and then the lysates were subjected to immunoprecipitation and western blotting using indicated antibodies. **(C, D)** After serum-starvation for 24 hours, PC3 cells were treated with 100ng/ml of IGF-I for 5 minutes. The cell lysates were analyzed by immunoprecipitation and western blotting using indicated antibodies. **(E)** The schematic diagrams show the domain structure of USP9X and deletion mutants. **(F, G)** The plasmids expressing the deletion mutants of USP9X together with Myc-IRS-1 (F) or IRS-2 (G) were transfected into HEK293T cells. After serum-starvation for 24 hours, the lysates were immunoprecipitated with anti-FLAG antibody, followed by western blotting using indicated antibodies.

To investigate the mechanisms regulating IRS-2 protein stability, we focused on IRS-2-associated proteins. By liquid chromatography/tandem mass spectrometry (LC-MS/MS) analysis of the IRS-2-co-immunoprecipitated proteins, we identified a deubiquitinase USP9X as an IRS-2-associated protein. To confirm our results, FLAG-IRS-1 or FLAG-IRS-2 was transfected into HEK293 cells and cell lysates were immunoprecipitated with FLAG-agarose beads. Immunoblotting analysis of immunoprecipitates revealed that USP9X was co-immunoprecipitated both with FLAG-IRS-1 and IRS-2 (Figure 1A). In addition, USP9X interacted with endogenous IRS-1/2 in PC3 human prostate cancer cells (Figure 1B). Furthermore, the interaction between USP9X and IRS-1/2 was not dependent on IGF-I stimulation (Figures 1C and 1D).

We next generated a series of deletion mutants of USP9X and examined their binding to IRS-1/2. USP9X was divided into four regions, namely N1, N2, C1 and C2 (Figure 1E). Only the C1 region contains a characterized domain structure called Peptidase C-19 domain, which is a common catalytic domain found among USP family proteins [19]. The deletion mutants of USP9X were transfected together with Myc-IRS-1 or IRS-2 into HEK293T cells, and immunoblotting analysis revealed that only C1, but not other regions interacted both with IRS-1 and IRS-2 (Figures 1F, and 1G), indicating that the catalytic domain of USP9X is sufficient for its binding to IRS-1/2.

### USP9X knockdown decreased IRS-2 protein level through ubiquitin proteasomal degradation in PC3 cells

**Figure 2:**
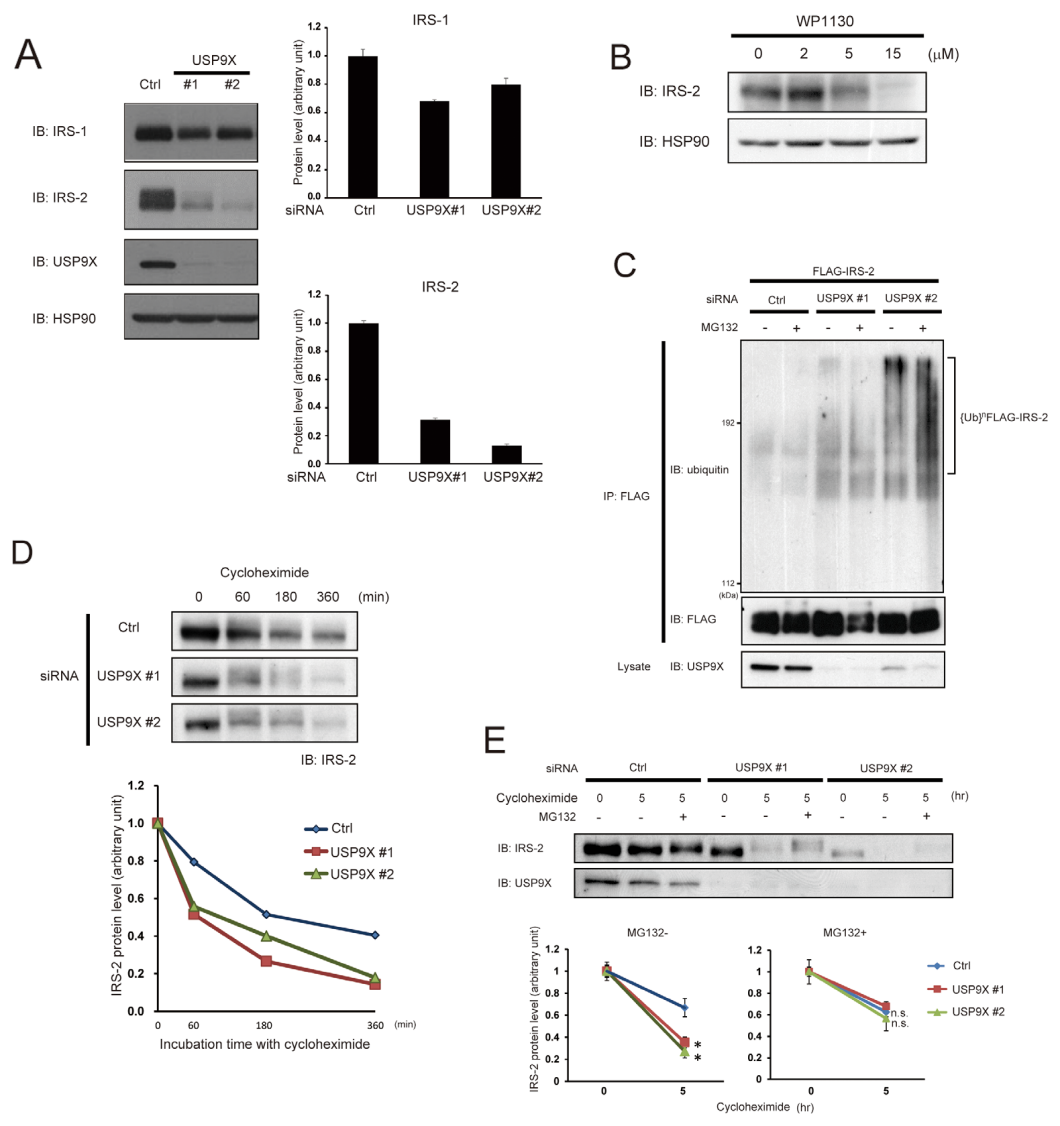
USP9X deubiquitinates IRS-2 to prevent its proteasomal degradation **(A)** PC3 cells were transfected with control siRNA or two different siRNAs targeting USP9X (#1 and #2), and cultured in the serum-free medium for 24 hours. The cell lysates were subjected to western blotting using indicated antibodies. HSP90 was used as loading control (left panel). This result is a representative of three independent experiments. The graphs represent the protein levels of IRS-1 and IRS-2 (right panel). **(B)** After serum-starvation for 22 hours, PC3 cells were treated with WP1130 for 2 hours. The cell lysates were analyzed by western blotting using indicated antibodies. HSP90 was used as internal control. **(C)** HEK293T cells were transfected with siRNA of USP9X or control. 24 hours after transfection, the cells were transfected with FLAG-IRS-2 and serum-starved for 20 hours followed by the treatment with MG132 (20 μM) for 2 hours. The cell lysates were denatured by boiled for 10 minutes in 1% SDS-containing buffer. FLAG-IRS-2 was immunoprecipitated by anti-FLAG antibody and the immunoprecipitates were analyzed by western blotting using indicated antibodies. **(D)** PC3 cells transfected with USP9X siRNA were serum-starved for 24 hours, and treated with cycloheximide (1 μg/ml) for the indicated time (top). The cell lysates were analyzed by western blotting using anti-IRS-2 antibody, and the protein levels of IRS-2 were normalized according to the levels at the time point of 0 hour (bottom). **(E)** PC3 cells transfected with control siRNA or USP9X siRNA were serum-starved for 24 hours, and treated with cycloheximide (1 μg/ml) and MG132 (20 μM) for the indicated time. The cell lysates were subjected to western blotting using indicated antibodies (top). The protein levels of IRS-2 were normalized according to the levels at the time point of 0 hours (bottom).

To investigate the role of USP9X in the control of IRS stability, we next performed siRNA-mediated knockdown of USP9X and examined IRS-1/2 protein levels. As shown in Figure 2A, USP9X protein was successfully reduced relative to control. Knockdown of USP9X decreased the amount of both IRS-1/2, and the reduction was much more severe for IRS-2 (Figure 2A). Treatment with WP1130, which selectively inhibits several deubiquitinases including USP9X [20], also significantly reduced the amounts of IRS-2 in a dose-dependent manner (Figure 2B). Since USP9X is reported to deubiquitinate and stabilize its substrates, we next investigated whether the ubiquitination of IRS-2 is enhanced in USP9X-depleted cells. HEK293T cells were co-transfected with siRNA targeting USP9X and FLAG-IRS-2 plasmid, and treated with a proteasome inhibitor, MG132. Immuno-purified FLAG-IRS-2 protein was analyzed by western blotting using ubiquitin-specific antibody. As a result, a high molecular mass smear, typical for ubiquitinated proteins was strongly detected only in siUSP9X cells (Figure 2C), indicating that depletion of USP9X increased ubiquitination of IRS-2. We next performed cycloheximide chase assay to evaluate the degradation rate of IRS-2. As expected, in siUSP9X cells, the degradation rate of IRS-2 was significantly accelerated (Figure 2D). Moreover, such acceleration was canceled by MG132 treatment (Figure 2E). These data suggest that USP9X deubiquitinates IRS-2 and prevents its proteasomal degradation.

### USP9X knockdown decreased IGF-IR protein level

**Figure 3:**
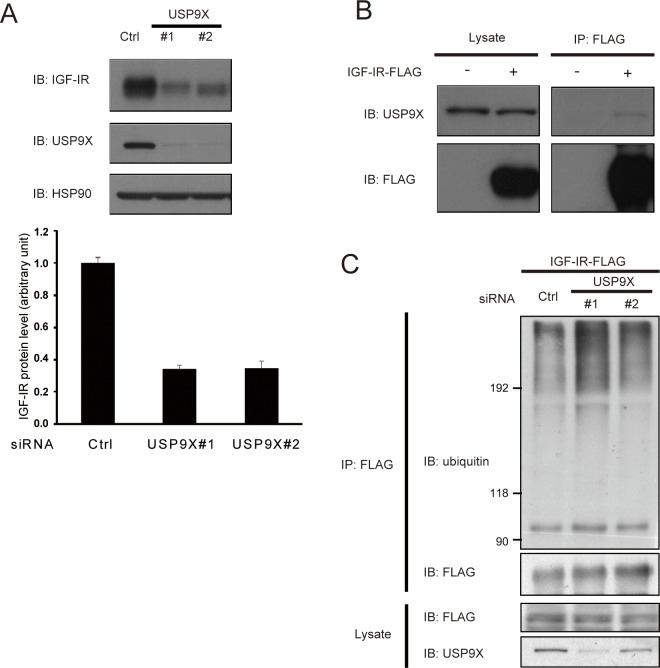
USP9X interacts with IGF-IR and stabilizes it **(A)** PC3 cells were transfected with control siRNA or USP9X siRNA, and serum-starved for 24 hours. The cells lysates were subjected to western blotting using indicated antibodies. HSP90 was used as internal control (top). This result is a representative of three independent experiments. The graph represents the protein level of IGF-IR (bottom). **(B)** HEK293T cells expressing IGF-IR-FLAG or FLAG alone were subjected to immunoprecipitation and western blotting using indicated antibodies. **(C)** HEK293T cells were transfected with control siRNA or USP9X siRNA. 24 hours after transfection, the cells were transfected with the plasmid expressing IGF-IR-FLAG and serum-starved for 20 hours. The cell lysate was denatured by boiling for 10 minutes in 1% SDS-containing buffer and analyzed by immunoprecipitation and western blotting using indicated antibodies.

In the process of investigating the effects of USP9X knockdown, we found that the amounts of IGF-IR as well as IRS-2 were significantly reduced in siUSP9X cells (Figure 3A). This result suggests that USP9X contributes to the stabilization of IGF-IR. In addition, co-immunoprecipitation analysis revealed that USP9X associated with exogenously expressed IGF-IR in HEK293T cells (Figure 3B). Furthermore, knockdown of USP9X significantly increased IGF-IR ubiquitination in HEK293T cells (Figure 3C). These results suggest that USP9X is required for IGF-IR deubiquitination and contributes to its stabilization.

### USP9X is required to maintain Erk1/2 phosphorylation through IRS-2 in PC3 cells

**Figure 4:**
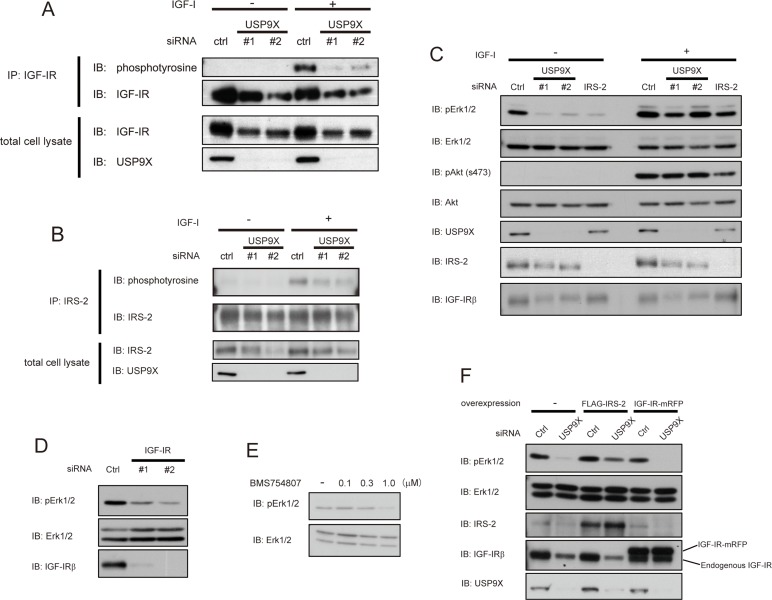
USP9X maintains the activation of Erk pathway by stabilizing IRS-2 **(A, B)** PC3 cells were transfected with control, USP9X siRNA, serum-starved for 24 hours, and stimulated with 100 ng/ml of IGF-I for 5 minutes. The cell lysates were subjected to immunoprecipitation and western blotting using indicated antibodies. **(C)** PC3 cells transfected with control, USP9X or IRS-2 siRNA were serum-starved for 24 hours, and stimulated with 100 ng/ml of IGF-I for 5 minutes. The cell lysates were subjected to western blotting analysis using indicated antibodies. **(D)** PC3 cells transfected with siRNA of IGF-IR or control were cultured in the serum-free medium for 24 hours. The lysates were analyzed by western blotting using indicated antibodies. **(E)** PC3 cells were cultured in the serum-free medium containing BMS754807 at indicated concentrations for 24 hours. The cell lysates were subjected to western blotting using indicated antibodies. **(F)** PC3 cells stably expressing empty vector, FLAG-IRS-2 or IGF-IR-mRFP were transfected with control or USP9X siRNA and serum-starved for 24 hours. The cell lysates were analyzed by western blotting using indicated antibodies.

Since USP9X knockdown reduced two important mediators of IGF-I signaling, IRS-2 and IGF-IR in PC3 cells, we assessed the effect of knockdown of USP9X on IGF-I signaling. In PC3 cells, IGF-IR and IRS-2 were tyrosine phosphorylated in response to IGF-I stimulation (Figures 4A and 4B). Consistent with the decrease in protein level, knockdown of USP9X decreased tyrosine phosphorylation levels of IGF-IR (Figure 4A). The decrease in IRS-2 tyrosine phosphorylation by USP9X knockdown was greater than the decrease in protein level (Figure 4B), which may reflect the decreased IGF-IR tyrosine phosphorylation. Erk1/2 and Akt kinases are downstream signaling molecules of IRS proteins in the IGF-IR signaling cascade. Knockdown of USP9X significantly reduced Erk1/2 phosphorylation in PC3 cells especially in the absence of IGF-I stimulation, while Akt phosphorylation was not affected (Figure 4C). Knockdown of IRS-2 or IGF-IR (Figures 4C and 4D), or treatment with IGF-IR kinase inhibitor (Figure 4E) also suppressed Erk1/2 phosphorylation in the absence of IGF-I. These data indicate that Erk1/2 is constitutively phosphorylated in an IGF-IR-dependent manner even in the absence of IGF-I stimulation in PC3 cells, though tyrosine phosphorylation of IGF-IR and IRS-2 was not detected. Taken together, these data suggest that USP9X and the activation of the IGF-IR-IRS-2 axis are required for the constitutive phosphorylation of Erk1/2 in PC3 cells.

We next speculated that the reduction of Erk1/2 phosphorylation in siUSP9X cells might be caused by the deficiency of IRS-2 or IGF-IR. To test this possibility, we rescued the expression of either IRS-2 or IGF-IR by retroviral infection of FLAG-IRS-2 or IGF-IR-mRFP in siUSP9X cells, and assessed Erk1/2 phosphorylation. As a result, the reduction of Erk1/2 phosphorylation was restored only in IRS-2 rescued cells but not in IGF-IR rescued cells (Figure 4F). These results clearly indicated that the reduction of Erk1/2 phosphorylation in siUSP9X cells was a consequence of IRS-2 deficiency.

### USP9X contributes to DNA synthesis of PC3 cells by maintaining the quantity of IRS-2

**Figure 5:**
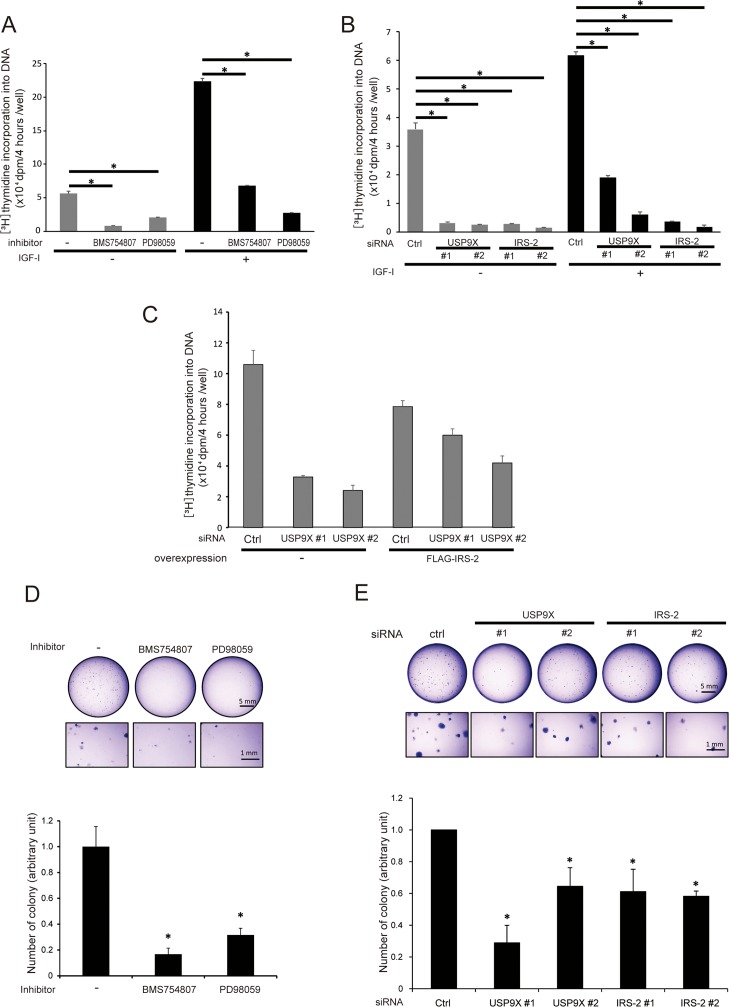
USP9X contributes to anchorage-dependent/independent cell growth induced by the activation of IGF-IR-IRS-2-Erk1/2 pathway **(A)** PC3 cells were cultured in the serum-free medium containing solvent control, 300 nM of BMS754807 or 100 μM of PD98059 for 48 hours. The cells were incubated with or without 100 ng/ml of IGF-I for 20 hours. For the last 4 hours, we added [^3^H]-thymidine to the medium, and measured the thymidine incorporation into DNA. **(B)** PC3 cells were transfected with control, USP9X or IRS-2 siRNA. The cells were serum-starved for 48 hours and incubated with or without 100 ng/ml of IGF-I for 20 hours. For the last 4 hours, we added [^3^H]-thymidine to the medium, and measured the thymidine incorporation into DNA. **(C)** PC3 cells stably expressing empty vector or FLAG-IRS-2 were transfected with siRNA of USP9X or control and serum-starved for 44 hours. For the last 4 hours, we added [^3^H]-thymidine to the medium, and measured the thymidine incorporation into DNA. **(D)** PC3 cells were resuspended in 0.32 % agarose containing medium with solvent control, 100 nM of BMS754807 or 50 μM of PD98059, and cultured for 13 days. The formed colonies were visualized by crystal violet staining (top), and the number of colonies was normalized according to that of control dish (bottom). **(E)** PC3 cells were transfected with siRNA of control, USP9X or IRS-2, and resuspended in 0.32% agarose containing medium. After culture for 13 days, the formed colonies were visualized by crystal violet staining (top). The number of colonies was normalized according to that of control dish (bottom).

Since the Erk1/2 pathway plays critical roles in induction of cell proliferation, we investigated the effects of USP9X knockdown on DNA synthesis in PC3 cells by measuring thymidine incorporation into DNA. Even in the absence of IGF-I stimulation, a substantial level of DNA synthesis was observed, and IGF-I-stimulation increased DNA synthesis approximately two- to four-fold in control cells (Figures 5A and 5B). Pharmacological inhibition of IGF-IR kinase and MEK reduced the DNA synthesis irrelevant to IGF-I stimulation (Figure 5A), suggesting that the phosphorylation of IGF-IR and Erk1/2 are critical for DNA synthesis of PC3 cells both in the absence and presence of IGF-I. In addition, consistent with the phosphorylation status of Erk1/2, knockdown of either USP9X or IRS-2 also significantly reduced DNA synthesis in the absence of IGF-I (Figure 5B left). In addition, IRS-2 overexpression partially restored the reduction of DNA synthesis in USP9X knocked down cells (Figure 5C). These results showed that USP9X contributes to DNA synthesis in PC3 cells through the maintenance of IRS-2 quantity and thereby Erk1/2 phosphorylation.

### USP9X contributes to anchorage-independent growth of PC3 cells

Anchorage-independent growth is an indicator of tumor growth. To investigate the role of USP9X on the malignant phenotype of PC3 cells, we performed soft-agar colony formation assay. The inhibition of either IGF-IR kinase or MEK significantly decreased the number of colonies compared to control treatment (Figure 5D), suggesting that IGF-IR kinase and Erk1/2 activity are critical for anchorage-independent growth of PC3 cells. Furthermore, knockdown of either USP9X or IRS-2 also significantly reduced the number of colonies (Figure 5E). Taken together, these results demonstrated that in PC3 cells, USP9X contributes to anchorage-independent growth by maintaining the IGF-IR-IRS-2-Erk1/2 axis.

### USP9X does not interact with IRS-2 in LNCaP cells

LNCaP is another prostate cancer cell line which shows relatively indolent behavior [21]. In LNCaP cells, mRNA expression of both USP9X and IRS-2 was significantly higher than PC3 cells, while protein levels of USP9X and IRS-2 were slightly lower and markedly lower than PC3, respectively (Supplementary Figure 2A and 2B). These results suggest that USP9X and IRS-2 protein levels are not controlled by the levels of gene expression. In addition, USP9X in LNCaP cells was not co-immunoprecipitated with IRS-2 (Supplementary Figure 1), suggesting that USP9X does not deubiquitinate IRS-2 in LNCaP cells.

## DISCUSSION

In the present study, we identified a deubiquitinase, USP9X as a novel IRS-associated protein, and elucidated its physiological roles in control of IGF signaling and growth of PC3 human prostate cancer cells.

USP9X is a deubiquitinase, which belongs to the USP family, and has been reported to regulate numerous physiological pathways such as transforming growth factor (TGF) β, Wnt and apoptosis signaling pathways [19, 22–26]. So far, it was uncertain whether USP9X has a role in regulation of IGF signaling pathway. In this study, our findings provided the first evidences that USP9X regulates the protein level of IRS-2, and therefore regulates the IGF-IR/IRS-2/Erk1/2 axis in PC3 human prostate cancer cells.

In general, ubiquitination of receptor tyrosine kinases (RTKs) causes their endocytosis, followed by the sorting to either recycling or degradation by lysosome and proteasome [27–30]. In addition, it has been reported that USP8-mediated deubiquitiantion of one RTK, epidermal growth factor receptor (EGFR) promotes its degradation [31, 32]. Another deubiquitinase, Associated molecule with the SH3 domain of STAM, AMSH is also implicated in EGFR degradation [33]. These reports indicate that deubiquitination as well as ubiquitination has roles in determining the fate of RTKs. We found that USP9X interacted with IGF-IR, and that knockdown of USP9X decreases IGF-IR protein level and increased ubiquitination of IGF-IR (Figures 3A, 3B, and 3C). These results raise the possibility that USP9X deubiquitinates IGF-IR to prevent its degradation.

USP9X has only one characterized domain structure, the Peptidase C19 domain. We found that both IRS-1/2 interacted with this domain of USP9X (Figure 1G). Since several substrates of USP9X, such as MCL-1 and β-Catenin are reported to interact with the Peptidase C19 domain of USP9X [5, 23], we speculated that both IRS-1 and 2 are substrates of USP9X. Knockdown of USP9X dramatically reduced IRS-2 protein level, increased IRS-2 ubiquitination, and promoted the proteasomal degradation of IRS-2 in PC3 cells (Figures 2A, 2C, and 2E), suggesting that USP9X also deubiquitinates IRS-2 to prevent its proteasomal degradation. On the other hand, the reduction of IRS-1 protein level was relatively small, suggesting that deubiquitination of IRS-1 by USP9X is not so active as for IRS-2. The distributions of lysine residues are poorly conserved between IRS-1 and 2. In addition, it has been reported that E3 ligase, CUL7 ubiquitinates only IRS-1 but not IRS-2 in response to IGF-I stimulation [16]. We have also reported that another E3 ligase Nedd4 upregulates tyrosine phosphorylation of only IRS-2 by mono-ubiquitination [34]. These indicate that the ubiquitinated sites and ubiquitin linkage types of IRS-1 and 2 are different from each other. Thus, it is possible that USP9X preferentially deubiquitinates IRS-2 much more than IRS-1 because of the difference of such ubiquitination patterns.

Erk1/2 and Akt are major downstream kinases of the IGF signaling pathways. Due to their proliferative effects, these pathways are thought to be central players in the progression of many types of cancer. In PC3 cells, Erk1/2 was constitutively phosphorylated even without IGF-I stimulation (Figure 4C), though tyrosine phosphorylation of IGF-IR and IRS-2 was merely detected under the same conditions (Figures 4A and 4B). Pharmacological inhibition of IGF-IR kinase or knockdown of IGF-IR or IRS-2 reduced the phosphorylation of Erk1/2 (Figures 4D and 4E). These results suggest that even without IGF-I stimulation, Erk1/2 is phosphorylated by the IGF-IR-IRS-2 pathway activation, which would be at a level too low to detect. Knockdown of USP9X significantly reduced Erk1/2 phosphorylation without IGF-I stimulation (Figure 4C), and the reduction was restored by overexpression of IRS-2, but not by IGF-IR (Figure 4F). These results indicate that the high expression of IRS-2 that is maintained by USP9X is necessary for Erk1/2 activation, and that as long as IRS-2 expression is high, relatively small amount of IGF-IR is enough to phosphorylate Erk1/2. On the other hand, Akt kinase was not phosphorylated in the absence of IGF-I, suggesting that the activation level of IGF-IR kinase without IGF-I stimulation is not enough to phosphorylate Akt kinase. Autocrine/paracrine modes of regulation are reported to promote proliferation of several cancers [35–37]. In addition, PC3 cells were shown to be capable of slow proliferation in the absence of added growth factors by secreting a small amount of IGF-II which binds to and activates IGF-IR [38]. Taken together, in PC3 cells, IGF-IR is constantly activated at a low level by autocrine/paracrine mechanisms. Owing to the high expression, IRS-2 can amplify the signaling effect of IGFs, resulting in the activation of the Erk1/2 pathway.

Consistent with the phosphorylation status of Erk1/2, substantial DNA synthesis was observed even in the absence of IGF-I (Figures 5A and 5B). Pharmacological inhibition of IGF-IR kinase and Erk1/2 pathway severely inhibited DNA synthesis in PC3 cells (Figure 5A), indicating that the proliferation of PC3 cells is highly dependent on the activation of IGF-IR kinase and Erk1/2 activation. It is also consistent with the phosphorylation status of Erk1/2, knockdown of USP9X or IRS-2 decreased DNA synthesis (Figure 5B left), and IRS-2 overexpression reserved the decrease in USP9X knocked down PC3 cells (Figure 5C). By these results, we concluded that USP9X contributes to proliferation of PC3 cells through maintenance of IRS-2-Erk1/2 axis.

Under IGF-I stimulated conditions, both Erk1/2 and Akt kinase were phosphorylated, and the knockdown of USP9X and IRS-2 barely affected the phosphorylation of both proteins (Figure 4C). It is well known that another insulin receptor substrate protein, Shc also mediates the activation of IGF-IR kinase to Erk1/2. In addition, we have previously reported that PI3K is directly bound to IGF-IR and activated [39]. Combined with these reports, our results suggest that when IGF-IR is strongly activated, Erk1/2 and Akt kinases are phosphorylated independently of IRS-2. Further studies are needed in order to understand the reason why the phosphorylation of Erk1/2 is highly dependent on IRS-2 only in the absence of IGF-I.

Although the activation of Erk1/2 was merely affected by knockdown of either USP9X or IRS-2, DNA synthesis was significantly decreased (Figure 5B right). These results suggest that the activation of Erk1/2 is necessary but not sufficient for DNA synthesis of PC3 cells. It seems that USP9X and IRS-2 themselves are also necessary for DNA synthesis. Further investigation is required to understand the regulatory mechanisms that control the proliferation of PC3 cells.

Anchorage-independent growth is a critical step in the acquisition of malignancy [40]. PC3 cells are known to have the ability for anchorage-independent growth [41]. We revealed that anchorage-independent growth of PC3 cells was significantly impaired by inhibition of either IGF-IR kinase or Erk1/2 pathway (Figure 5D). In addition, knockdown of USP9X and IRS-2 also impaired it (Figure 5E). Taken all together, these results suggest that the maintenance of IRS-2 quantity by USP9X and therefore the maintenance of constitutive activation of Erk1/2 contribute to the malignant growth of PC3 cells.

The growth of prostate cancer is highly dependent on IGF [9–11]. In the present study, we revealed that the IGF-IR/IRS-2 pathway, which requires a high level of IRS-2 protein, is critical for anchorage-independent growth in human prostate cancer cell line PC3. On the other hand, in another prostate cancer cell line LNCaP, IRS-2 protein level was too low to detect (Supplementary Figure 2B), suggesting that the dependency on IGF-IR/IRS-2 pathway is lower in LNCaP than PC3. LNCaP is an androgen-dependent prostate cancer and usually used as a relatively indolent form of prostate cancer, while PC3 is a castration-resistant and highly aggressive form of prostate cancer. Taken together, IRS-2 protein may be up-regulated preferentially in highly malignant types of prostate cancer, and therefore the dependency on IGF-IR/IRS-2 pathway may be high especially in such type of prostate cancer.

The mRNA expression of both USP9X and IRS-2 was significantly higher in LNCaP than PC3, however, the protein level of USP9X and IRS-2 was slightly less and was dramatically less than PC3, respectively (Supplementary Figure 2A and 2B). These observations suggest that USP9X and IRS-2 protein level is regulated by the control of protein stability rather than mRNA expression. This may explain the reason why Oncomine analysis failed to show any change of these gene expression between normal and prostate cancer tissue (data not shown) [42]. In LNCaP cells, IRS-2 protein level was dramatically low, while substantial level of USP9X protein exist in LNCaP (Supplementary Figure 2B). In addition, the interaction between USP9X and IRS-2 was not observed in LNCaP cells (Supplementary Figure 1), suggesting that USP9X in LNCaP does not deubiquitinate and stabilize IRS-2 because of the absence of interaction between them. Although the Human Protein Atlas database showed IRS-2 protein level was not different between normal and prostate cancer tissue (data not shown) [43], it has been reported that in a portion of human prostate cancer tissue, IRS-2 protein level is up-regulated [8]. Thus, it is likely that at least in a portion of human prostate cancer, USP9X interacts with IRS-2 resulting in its deubiquitination and stabilization, followed by the maintenance of high protein level of IRS-2. In addition to prostate cancer, previous reports have shown that in human endometrial cancer, leiomyosarcoma and several cell lines of various human cancer, IRS-2 protein level is up-regulated [8, 44]. It is true that the protein up-regulation of IRS-2 may be caused by several steps other than protein stabilization such as the promotion of gene expression or translation, but at least in a portion of these IRS-2-high-type cancers, stabilization of IRS-2 protein by USP9X may contribute to the maintenance of high protein level of IRS-2. The mechanisms underlying stabilization of USP9X protein are yet unclear. Further studies are required to understand it.

In summary, USP9X was required for the maintenance of IRS-2 high expression in PC3 cells. The high expression of IRS-2 was required for the constitutive activation of the IGF-IR-IRS-2-Erk1/2 axis which was critical for anchorage-independent growth of PC3 cells. These findings provide new insights into how USP9X controls IRS-2 protein level and how IRS-2 high expression contributes to cancer attributes in prostate cancer. Our results provide a rationale to develop a USP9X-IRS-2 binding inhibitor as a cancer therapy.

## MATERIALS AND METHODS

### Cell culture

Human embryonic kidney (HEK) 293 cells (kind gifts from Dr. Koichi Suzuki (National Institute of Infectious Diseases, Tokyo, Japan)), HEK293T cells and prostate cancer PC-3 cells (kind gifts from Dr. Akio Matsubara (Hiroshima University, Hiroshima, Japan)) were cultured as described previously [34]. LNCaP cells (kind gifts from Dr. Eijiro Nakamura (Kyoto University, Kyoto, Japan)) were cultured as described previously [45, 46].

### Antibodies and reagents

The antibodies and reagents used in this study were obtained from the following sources. Anti-IRS-1 antibody and anti-IRS-2 antibody for immunoprecipitation were raised in rabbits as described previously [47]. Anti-IRS-2 antibody for immunoblotting, anti-IGF-IR β antibody, anti-HSP90 α/β antibody and anti-ubiquitin antibody (P4D1) were from Santa Cruz Biotechnology (Santa Cruz, CA, USA). Anti-USP9X antibody was kindly provided by Dr. Stephen A. Wood (Griffith University, Queensland, Australia). Anti-USP9X antibody was also obtained from Abcam (Cambridge, MA, USA). Anti-FLAG antibody and anti-phosphotyrosine antibody (4G10) were from Sigma (St. Louis, MO, USA). Anti-Myc antibody (9E10) was from Millipore (Billerica, MA, USA). Anti-Akt antibody, anti-pAkt (Ser473) antibody, anti-Erk1/2 antibody, anti-pErk1/2 antibody and anti-IGF-IR β antibody were from Cell Signaling Technology (Dancers, MA, USA). MG132 was from Cell Signaling Technology (Dancers, MA, USA). Cycloheximide was from Nacalai Tesque (Tokyo, Japan). PD98059 was from Sigma (St. Louis, MO, USA), BMS754807 was from Funakoshi (Tokyo, Japan). WP1130 was from Life Sensors (Malvern, PA, USA). Recombinant human IGF-I was kindly gifted by Dr Toshiaki Ohkuma (Fujisawa Pharmaceutical Co., Osaka, current Astellas Pharma Inc., Tokyo, Japan).

### Plasmids and siRNAs

pMyc-IRS-1, pFLAG-IRS-1, pFLAG-IRS-2, pMyc-IRS-2-FLAG or pIGF-IR-FLAG were constructed as described previously [34]. The plasmids expressing deletion mutants of rat IRS-1, human IRS-2 or USP9X were cloned into pFLAG-4-5. Small interfering RNAs (siRNAs) used in this study were obtained from Nippon Gene Material Corp. (Tokyo, Japan). The siRNAs comprised the following sequences: USP9X #1; 5′-AGACUGGUUUCCACUUUUA-3′, USP9X #2; 5′-GAUGAGGAACCUGCAUUUC-3′, IRS-2 #1 5′-UCGGCUUCGUGAAGCUCAAdTdT-3′, IRS-2 #2; 5′-GGCUGAGCCUCAUGGAGCAdTdT-3′, IGF-IR #1; 5′-GGAGUUCAAUUGUCACCAUdTdT-3′, IGF-IR #2; 5′-GGAUUGAGUUUCUCAACGAdTdT-3′. For retroviral expression, FLAG-tagged IRS-2 and mRFP-tagged IGF-IR were cloned into pMXs-puro. Full length of the mouse cationic amino acid transporter (mCAT)-1 was cloned into pcDNA3.1.

### Transfection of plasmids and siRNA

The expression plasmids were transfected into HEK293T cells and PC3 cells using Polyethylenimine (PEI) as described previously [34]. The plasmids were transfected into HEK293 cells using Lipofectamine 2000 (Life technologies, Tokyo, Japan) by forward transfection method following the manufacturer's protocol. The siRNAs were transfected to HEK293T cells or PC3 cells using Lipofectamine RNAiMAX (Life Technologies, Tokyo, Japan) by the reverse transfection method following the manufacturer's protocol.

### Retrovirus production, infection and isolation of stable transfectant

Retrovirus production was performed as described previously [48]. PC3 cells were transfected pmCAT-1 24 hours before infection, and were infected the retroviruses as described previously. Infected cells were selected with puromycin.

### Immunoprecipitation and immunoblotting analysis

Cells were serum starved for 20-24 hours with Dulbecco's modified Eagle medium (DMEM) supplemented with 0.1% bovine serum albumin (BSA), and then incubated with or without human recombinant IGF-I (100 ng/ml) for 5 min. Cell lysates were prepared with lysis buffer (50 mM Tris-HCl, pH 7.4, 1 mM EDTA, 1 mM EGTA, 150 mM NaCl, 50 mM NaF, 1% Triton X-100, 100 kallikrein-inactivating units/ml aprotinin, 20 mg/ml phenylmethanesulfonyl fluoride, 10 mg/ml leupeptin, 5 mg/ml pepstatin) and subjected to immunoprecipitation and immunoblotting as described previously [34]. The concentration of each protein was quantified by ImageJ.

### Ubiquitination analysis

293T cells were transfected with the plasmids expressing FLAG-IRS-2 or IGF-IR-FLAG. Twenty four hours after transfection, the cells were serum-starved for 20-24 hours and then incubated with 20 μM of MG132 for 2 hours. The cells were harvested in lysis buffer supplemented with 2 mM N-ethylmaleimide, and the lysates were centrifuged at 15,000 × g for 10 min. The supernatant was mixed with 1% SDS and boiled for 10 min. The lysates were diluted 20 times with lysis buffer immediately after boiling, and then incubated on ice for 30 min, followed by immunoprecipitation with anti-FLAG antibody and immunoblotting using anti-ubiquitin antibody.

### DNA synthesis assay

Cells were serum starved for 48 hours, and then incubated with or without human recombinant IGF-I (100 ng/ml) for 20 hours. DNA synthesis was measured by using [^3^H] thymidine as described previously [48].

### Soft-agar colony formation assay

Soft-agar colony formation assay was performed using 12-well plates by the following procedure. Each well was plated with 500 μl of bottom medium (DMEM containing 10% FBS, 0.36% low melting agarose (Sigma, St. Louis, MO, USA)). The plates were incubated at 4°C for 20 min. 1.0×10^3^ of PC3 cells were mixed thoroughly with 500 μl of growth medium containing 0.36% low melting agarose, and plated onto a layer of 0.75 % agarose-containing medium, and then incubated at 4°C for 20 min. The cells were cultured for 13 days at 37°C. The cells were incubated with fixing solution (10% acetic acid, 10% methanol) on ice for 10 min, stained with 0.01% crystal violet on ice for 10 min, and washed with cold DW for 5 times. The colonies stained with crystal violet were counted using ImageJ.

### Quantitative RT-PCR analysis

Total RNA from PC3 cells and LNCaP cells were extracted with TRIzol reagent (Invitrogen, Tokyo, Japan) from six independently collected cells, respectively. Quantitative PCR was performed with THUNDERBIRD SYBR qPCR Mix (TOYOBO, Osaka, Japan) on an ABI StepOnePlus Real Time PCR System (Applied Biosystems). To normalize the relative expression, a standard curve was prepared for each gene for relative quantification, and the expression level of each gene was normalized to the β-actin gene. Specific primers for each gene were used: *IRS2* F; AGCTTCTTCTTCATCGAGGTG, *IRS2* R; AACTCGAAGAGCTCCTTGAG, *USP9X* F; GAATCCCATGACACAGATCAACC, *USP9X* R; CCTCATCAGATATCTGCTGAGCAAG, *ACTB* F; TTCCTTCCTGGGCATGGAG, *ACTB* R; GCAGTGATCTCCTTCTGCATC.

### Statistical analysis

The results shown are the mean ±SEM. Data were analyzed by one-way factorial ANOVA and Turkey-Kramer Post-hoc multiple comparison test. *P*<0.05 was considered statistically significant (shown as “^*^” in graphs).

## SUPPLEMENTARY MATERIALS FIGURES



## Abbreviations

IGF: insulin-like growth factor
IRS: insulin receptor substrate
USP9X: ubiquitin specific peptidase 9X
siRNA: small interference RNA
SH2: Src homology 2
PI3K: phosphatidylinositol 3-kinase
PIP3: phosphatidylinositol (3,4,5)-triphosphate
MAPK: mitogen-activated protein kinase
PEI: polyethylenimine
DMEM: Dulbecco's modified Eagle medium
BSA: bovine serum albumin
LC-MS/MS: liquid chromatography/tandem mass spectrometry
TGF: transforming growth factor
RTK: receptor tyrosine kinase
EGFR: epidermal growth factor receptor
