# Supplementary material for: IRS-2 deubiquitination by USP9X maintains anchorage-independent cell growth via Erk1/2 activation in prostate carcinoma cell line
Source: Oncotarget. 2018 Sep 21;9(74):33871–83. doi: 10.18632/oncotarget.26049 (PMC6188063; doi:10.18632/oncotarget.26049)
Supplement: Supplementary file 1 [file oncotarget-09-33871-s001.pdf]

IRS-2 deubiquitination by USP9X maintains anchorage-independent cell growth via Erk1/2 activation in prostate carcinoma cell line

SUPPLEMENTARY MATERIALS

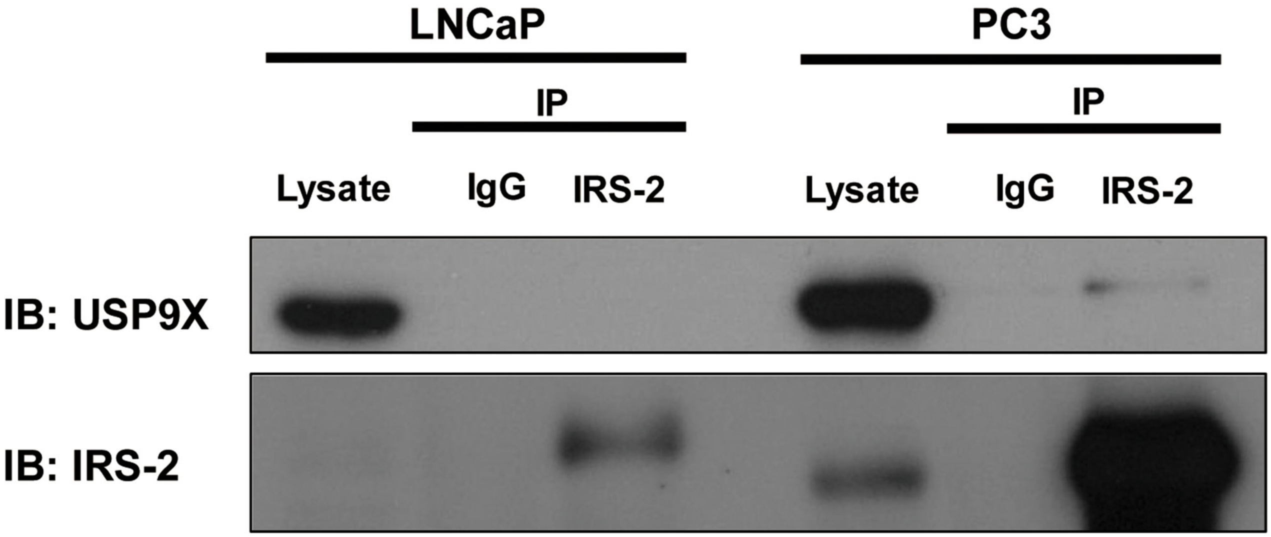

Supplementary Figure 1: USP9X does not interact with IRS-2 in LNCaP cells. PC3 cells and LNCaP cells were cultured under serum-free conditions for 24 hours and 4 hours, respectively. The cell lysates were immunoprecipitated with anti-IRS-2 antibody, followed by western blotting using indicated antibodies.

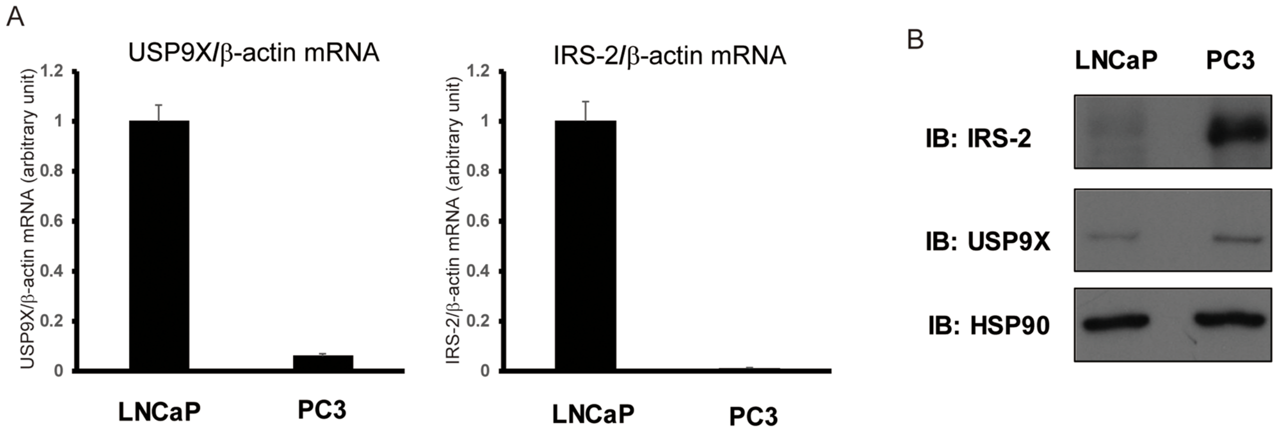

Supplementary Figure 2: IRS-2 protein level is lower in LNCaP than PC3. (A) Quantitative RT-PCR analysis of USP9X and IRS-2 genes from PC3 cells and LNCaP cells. Data are expressed as fold of the value of β-actin gene. **(B)** Lysates of PC3 cells and LNCaP cells were subjected to immunoprecipitation and western blotting using indicated antibodies.
